# A Timely Fable

**Published:** 1886-08

**Authors:** 


### *A TIMELY FABLE.*

From the German.—Respectfully dedicated to the "Seybert Commission."

There's a story by Krummacher,
  In my German reader told,
Of some erudite professors,
  Who were left out in the cold,
And the story hath a moral
  Apposite to young and old.

Now these excellent professors,
  Made a brotherly resolve
That, as voluntary testers
In their philosophic ways
  Their united heads would solve
Whence and what the hidden force is,
  That the magnet-needle sways,
  If on land or over seas,
Whatsoe'er one's shifting course is,
  Or the weather's changeful phase.

Thus resolved, these Doctors able,
  Built a vessel staunch and new.
Then upon the cabin's table,
For their equal observation,
  Rounded to a circle true,
Held the compass middle station;
And besides in handy reach,
Tomes and treatises that each
On occasion, fetched to use
In affirmance of his views.
Thus, with all things to their notion,
Out they sailed upon the ocean.

Then our wise investigators,
With their books and apparatus,
Round the compass held debate
As to nature's occult forces,
With their several ancient noses
Just above the radial plate,
Whilst the vessel good and stout,
Yon and hither sailed about.

But in consultation free,
Never two could quite agree,
What the slender bar controls
Into line between the poles.

One insisted 'twas a breath ;
One a current ever flowing,
Yet another equal knowing,
Signified that matter hath
Subtile elements that give it
Intimations of a spirit.
Thus they questioned and disputed,
Poopoo'd, pisched and hifaluted,
Every one his theory venting,
But no other one assenting;
Whilst the vessel, yon and hither
Drifted, all unnoticed whither.

Suddenly there came a shock,
And a crashing most terrific,
Then a craggy, scraggy rock,
Pierced the little craft and split it,
And the frightened college fellows
Dripping of the briny billows,
Only thought themselves to save
On the crags above the wave.

As the vessel rudely bursted,
Sank beneath the waters broad,
Our investigators, worsted,
Found for once, though strangely odd,
All their voices in accord,
That the compass was a fraud
And in no sort to be trusted.

---

**A Colorado cowboy** was recently bitten on the finger by a rattlesnake. He began to drink whisky as fast as possible, and had swallowed a gallon before it had the slightest effect on him. Then it began to get in its work, and the rattlesnake poison had no show. But the cowboy came near dying just the same.

